# Determinants of *Eimeria* and *Campylobacter* infection dynamics in UK domestic sheep: the role of co-infection

**DOI:** 10.1017/S0031182021000044

**Published:** 2021-04

**Authors:** Raed Taha Al-Neama, Kevin J. Bown, Damer P. Blake, Richard J. Birtles

**Affiliations:** 1 School of Science, Engineering and Environment, University of Salford, Manchester, M5 4WT, UK; 2 Department of Pathobiology and Population Sciences, The Royal Veterinary College, Hawkshead Lane, North Mymms, Hatfield, Hertfordshire, AL9 7TA, UK

**Keywords:** *Campylobacter*, co-infection, *Eimeria*, ovine

## Abstract

Coccidiosis caused by *Eimeria* species is a well-recognized disease of livestock. Enteric *Eimeria* infections are common, but disease usually only manifests when infection intensity is abnormally high. *Campylobacter* species are important zoonotic enteric bacterial pathogens for which livestock are important reservoir hosts. The diversity and epidemiology of ovine *Eimeria* and *Campylobacter* infections on two farms in north-western England were explored through a 24-month survey of shedding in sheep feces. Most animals were infected with at least one of 10 different *Eimeria* species, among which *E. bakuensis* and *E. ovinoidalis* were most common. An animal's age and the season of sampling were associated with the probability and intensity of *Eimeria* infection. Season of sampling was also associated with the probability of *Campylobacter* infection. Interestingly, higher intensities of *Eimeria* infections were significantly more common in animals not co-infected with *Campylobacter*. We explored the determinants of *E. bakuensis* and *E. ovinoidalis* infections, observing that being infected with either significantly increased the likelihood of infection with the other. The prevalence of *E. ovinoidalis* infections was significantly lower in sheep infected with *Campylobacter*. Recognition that co-infectors shape the dynamics of parasite infection is relevant to the design of effective infection control programmes.

## Graphical abstract

**Figure:**
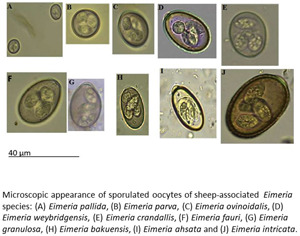

## Introduction

Coccidiosis of sheep is a frequently encountered disease caused by members of the apicomplexan genus *Eimeria* (Kaufmann, 1996). Coccidiosis manifests as diarrhoea, slow weight gain and occasionally death, and represents a significant economic burden to the sheep farming industry worldwide (Chartier and Paraud, 2012). Chronic asymptomatic *Eimeria* infections are extremely common; surveys around the world have shown that most individuals are infected and shed oocysts in their feces, typically in low numbers, thereby providing a constant source of infection for other animals (O'Callaghan *et al*., 1987; Dittmar *et al*., 2010). At least 15 different *Eimeria* species have been encountered in sheep (Kaufmann, 1996), although not all have been associated with coccidiosis. *Eimeria ovinoidalis*, *E. crandallis* and, to a lesser extent, *E. bakuensis*, *E. parva* and *E. ahsata* are more frequently linked with the disease (Skirnisson, 2007) than other species. The relative frequencies with which infection by different *Eimeria* species is encountered vary; *E. crandallis* and *E. parva* appear to be the most abundant species worldwide (Gul and Deger, 2002), although *E. weybridgensis*, *E. ovinoidalis* and *E. bakuensis* are also common in Europe (Reeg *et al*., 2005), but mixed infections by two or more *Eimeria* species are the norm (Arslan *et al*., 1999). Epidemiological surveys have identified numerous risk factors for *Eimeria* infection including animal age, with more infections in younger individuals, and season, with a higher prevalence of infection during wetter periods of year (de Souza *et al*., 2015). An individual's sex may also be influential (de Souza *et al*., 2015) as is their physiological status; detectable levels of shedding are more common during pregnancy (Dittmar *et al*., 2010). Furthermore, correlates with infection intensity have also been reported, with more intense infections in young animals (Reeg *et al*., 2005).

Campylobacter is the most frequently reported agent of bacterial gastroenteritis in many developed countries including the UK, where over 50 000 laboratory-confirmed cases are reported each year (PHE, 2018). Campylobacteriosis is a zoonosis and ruminant livestock are an important source of human infections (Thépault *et al*., 2017). Surveys in the UK have revealed a prevalence of infection of up to about 50% of grazing animals (Stanley *et al*., 1998; Jones, 2001; Grove-White *et al*., 2010; Sproston *et al*., 2011). Epidemiological studies have identified various risk factors for *Campylobacter* infection in sheep including a higher prevalence associated with increased stocking density and pasture quality, and a season pattern, with a peak prevalence of infections coinciding with lambing, weaning and the summer months (Stanley *et al*., 1998; Jones., 2001; Grove-White *et al*., 2010; Sproston *et al*., 2011).

*Eimeria* and *Campylobacter* represent two of a broad diversity of parasitic taxa associated with sheep that includes viruses, bacteria and eukaryotes such as protists, helminths and arthropods. Studies in sheep and other vertebrate hosts have demonstrated that co-infecting parasites often interact thereby shaping susceptibility to, and dynamics of, infection by each parasite (Craig *et al*., 2008; Telfer *et al*., 2008; Salgame *et al*., 2013; Leung *et al*., 2018). These co-infections may have specific veterinary and food safety implications (Thumbi *et al*., 2014; Van Wyk *et al*., 2014) and, ecologically, have consequences for host condition and fitness (Telfer *et al*., 2010). The impact of co-infectors on ovine *Eimeria* infections has yet to be assessed, but studies in other vertebrates have indicated *Eimeria* interacts with other parasites and influence their infection dynamics (Knowles *et al*., 2013; Mason *et al*., 2015) and host response (Zhang *et al*., 2018).

In this study, we used longitudinal monitoring of farmed sheep flocks in north-west England to determine the seasonal dynamics of *Eimeria* and *Campylobacter* infections and to explore the biotic and abiotic determinants of these.

## Materials and methods

### Sheep surveys

Surveys were carried out on two sheep farms, stock from both of which were grazed on Bethecar Moor, an extensive tract of common land (approximately 550 hectares) in southern Cumbria (north-west England). Farm A maintained a flock of approximately 600 Swaledale sheep which spent most of the year (about 300 days) on the Moor and were herded only five times per annum for brief periods (Table 1). Farm B maintained a flock of about 200 sheep, the majority of which were Texel but some Swaledale too. These animals were more intensively managed than those on Farm A, being herded far more regularly and spending markedly more time (about 160 days) on pasture around the farm (Table 1).

**Table 1.** Details of husbandry practices on study farms

Farm	Month (weeks off moor)	Reason	Parasite/pathogen control measures employed
A	November (3)	Mating	*Clostridium/Pasteurella* vaccination, application of anthelmintic
	January (1)	Pregnancy check	Application of ectoparasiticide
	March (3)	Lambing	Louping ill virus vaccination (young ewes), application of endectocides
	July (2)	Sheering	None
	September (1)	Removal of male lambs	*Clostridium/Pasteurella* vaccination, application of anthelmintic and ectoparasiticide
B	November (6)	Mating	Application of anthelmintic
	January (1)	Pregnancy check	None
	March/April (9)	Lambing	*Clostridium/Pasteurella* vaccination, application of anthelmintic and ectoparasiticide
	May (1)	Infection control	Application of anthelmintic
	June (1)	Infection control	Application of anthelmintic
	July (1)	Infection control	Application of anthelmintic
	August (1)	Infection control	Application of anthelmintic
	September (2)	Removal of male lambs	Application of anthelmintic
	October (1)	Infection control	Application of anthelmintic

On Farm A, sheep were surveyed on 11 occasions between September 2014 and September 2016. Each survey corresponded to the events described above bar March 2015. On Farm B, sheep were surveyed on 12 occasions between July 2015 and September 2016 (roughly once a month bar December 2015, January 2016 and June 2016). On each visit to each farm, freshly voided fecal samples were collected between 20 and 60 opportunistically selected sheep. Approximately 5 g of feces were collected into a 60 mL collection pot which was then immediately placed on ice held in a cold box.

The age of each sheep was estimated as being either <12, between 12 and 24, or >24 months on the basis of its age, size and condition on the advice of and with the help of farmers/experienced farm hands.

### Isolation, quantification and identification of *Eimeria* species

About 4 g of feces were added to 56 mL sterile distilled water and repeatedly mixed until a homogeneous suspension was obtained, which was then transferred to a sterile container through a tea-strainer. Ten microlitres of this filtrate were centrifuged at 172 × *g* for 5 min then the supernatant discarded, and the pellet resuspended in 4 mL of flotation fluid (saturated NaCl solution containing 500 g per litre of glucose). In total, 50 *μ*L of this suspension were transferred to two chambers of a McMaster slide then left for 5 min to allow any oocysts in the suspension to rise to the surface. Oocysts were counted by observation under 100 × magnification and standardized to oocysts per 1 g of feces.

Any sample in which *Eimeria* oocysts were observed was subjected to an induced sporulation process. One volume of sheep feces was resuspended in two volumes of 2.5% (w/v) potassium dichromate solution, sieved to remove coarse material then poured as a thin layer into a petri dish. These oocyst suspensions were then held for 10 days at room temperature to allow sporulation to occur. After this period, sporulated oocysts were pelleted by centrifugation at 1077 × *g* for 5 min then resuspended in flotation fluid such that a meniscus is formed at the top of the centrifuge tube. A cover slide was placed on the top of the centrifuge tube and left for 5 min, then carefully removed and observed under 100× magnification. The identity of oocysts present was determined by reference to a taxonomic key (Eckert *et al*., 1995).

### Isolation and identification of *Campylobacter* species

In total, 0.5 g of fresh (<24 h since collection) fecal material were suspended in 5 mL modified Preston broth (Oxoid, Basingstoke, Hampshire, UK) by vortexing. The suspension was incubated at 42°C for 24 h in a microaerophilic atmosphere, then 100 *μ*L of this suspension was spread onto charcoal cefoperazone deoxycholate agar agar (Oxoid). Plates were incubated at 42°C for 48 h in a microaerophilic atmosphere after which plates were examined for growth. Putative *Campylobacter* isolates were identified on the basis of colonial morphology and Gram staining. In order to confirm the identity of these isolates, boiled suspensions of colonies were incorporated as a template into a *Campylobacter* genus-specific PCR (Linton *et al*., 1997). A subset of 20 amplicons obtained from samples collected on Farm A were further characterized using Sanger sequencing, and all samples from Farm B that yielded an amplicon were retested using additional *Campylobacter*-specific PCRs (Yang *et al*., 2004; Vondrakova *et al*., 2014).

### Statistical analysis

All statistical analyses were performed using R software version 3.4 (R Development Core Team, 2015). To investigate factors that influenced an individual sheep's probability of being infected with *Eimeria* and/or *Campylobacter* species, generalized linear models (GLMs) were used that assumed a binomial error term and a logit link. Factors considered included age, season (spring = months March–May, summer = June–August, autumn = September–November, winter = December–February), farm, weather (rainfall/temperature) and co-infection. In addition to considering infection at the genus level, this method was also employed for the two most common *Eimeria* species, *E. ovinoidalis* and *E. bakuensis.* To investigate whether the potential non-independence of samples from the same farm was important, generalized linear mixed models with a binomial error term and a logit link were employed where farm was included as a random effect

To investigate factors that influenced the intensity of *Eimeria* infections, GLMs with a negative binomial error term and a log link were employed. The same factors as listed above were considered. Model selection was based on a backward stepwise model selection with variables dropped according to *P* value, with only those variables significant at the *P* < 0.05 level being retained in the final model.

## Results

### Epidemiological patterns

A total of 360 fecal samples were collected from sheep on Farm A. Of these, 350 were tested for the presence of *Eimeria* oocysts and 353 were tested for the presence of *Campylobacter* (Table 2). In total, *Eimeria* oocysts were present in 281 (80.0%) samples, with the prevalence of fecal shedding ranging from 66.7 to 97.6% between surveys and a mean intensity ranging from 61 to 2672 oocysts per gram of feces between surveys. The overall prevalence of *Campylobacter* infections, as determined by PCR, was 32.6% (115/353), with a range of 21.9–63.2% between surveys (Table 2). Sanger sequencing of 20 PCR amplicons confirmed them all to be derived from *Campylobacter* species.

**Table 2.** Prevalence and intensity of *Eimeria* infections, and prevalence of *Campylobacter* infections on Farm A

Survey	Survey date	No of *Eimeria*-positive samples/No of samples (%)	Mean No of *Eimeria* oocysts g^−1^ feces (s.e.)	No of *Campylobacter-*positive samples/No of samples (%)
1	16/09/2014	24/35 (68.6)	927 (325)	10/41 (24.4)
2	10/11/2014	13/19 (68.4)	325 (91)	7/20 (35.0)
3	22/01/2015	22/28 (78.6)	212 (31)	10/30 (33.3)
4	18/02/2015	40/41 (97.6)	135 (19)	24/38 (63.2)
5	23/07/2015	54/62 (87.1)	1203 (274)	17/60 (28.3)
6	11/09/2015	28/33 (84.8)	285 (71)	7/32 (21.9)
7	09/11/2015	14/18 (77.8)	106 (25)	3/19 (15.8)
8	24/02/2015	20/29 (69.0)	253 (102)	12/29 (41.4)
9	09/05/2015	21/23 (91.3)	294 (60)	7/23 (30.4)
10	25/07/2015	25/32 (78.1)	2676 (502)	7/31 (22.6)
11	12/09/2015	20/30 (66.7)	61 (9)	11/30 (36.7)

s.e., standard error.

A total of 440 fecal samples were collected from Farm B. Of these, 433 were tested for the presence of *Eimeria* oocysts and 430 were tested for the presence of *Campylobacter* (Table 3). In total, *Eimeria* oocysts were present in 327 (75.5%) samples, with a prevalence of infection ranging from 53.3 to 100% between surveys and a mean intensity ranging from 62 to 460 oocysts per gram of feces between surveys. The overall prevalence of *Campylobacter* infections, as determined by genus and a combination of species-specific PCRs, was 29.1% (125/430), with a range of 10.8–62.5% between surveys (Table 3).

**Table 3.** Prevalence and intensity of *Eimeria* infections, and prevalence of *Campylobacter* infections on Farm B.

Survey	Survey date	N^o^ of *Eimeria*-positive samples/No of samples (%)	Mean No of *Eimeria* oocysts g^−1^ feces (s.e.)	No of *Campylobacter-*positive samples/No of samples (%)
1	28/07/2015	41/41 (100.0)	460 (124)	25/40 (62.5)
2	20/08/2015	47/47 (100.0)	159 (39)	20/52 (38.5)
3	02/09/2015	38/55 (69.1)	180 (25)	15/55 (27.3)
4	30/10/2015	16/30 (53.3)	209 (75)	7/30 (23.3)
5	20/11/2015	14/23 (60.1)	86 (20)	5/23 (21.7)
6	04/02/2016	26/35 (74.3)	240 (70)	10/36 (27.8)
7	23/03/2016	24/33 (72.7)	329 (94)	13/34 (38.2)
8	27/04/2016	23/35 (65.7)	361 (84)	13/31 (41.9)
9	27/05/2016	36/42 (85.7)	451 (92)	14/37 (37.8)
10	08/07/2016	25/25 (100.0)	353 (70)	7/25 (28.0)
11	18/08/2016	17/30 (56.7)	240 (45)	7/30 (23.3)
12	22/09/2016	20/37 (54.1)	62 (8)	4/37 (10.8)

s.e., standard error.

The two most commonly encountered *Eimeria* species in this study were *E. ovinoidalis* and *E. bakuensis* (Table 4). Both species were frequently encountered in samples collected from both study farms. In total, *E. ovinoidalis* oocysts were present in 179 (51.1%) of samples collected on Farm A and 192 (44.3%) of samples collected on Farm B. *Eimeria bakuensis* oocysts were present in 136 (38.9%) of samples collected on Farm A and 198 (45.7%) of samples collected on Farm B (Table 4).

**Table 4.** Prevalence of *E. ovinoidalis* and *E. bakuensis* infections on Farms A and B

Farm	Survey	No of *E. ovinoidalis-*positive samples/No of samples (%)	No of *E. bakuensis-*positive samples/No of samples (%)
A	1	13/35 (37.1)	10/35 (28.6)
	2	9/19 (47.4)	8/19 (42.1)
	3	14/28 (50.0)	7/28 (25.0)
	4	20/41 (48.8)	18/41 (43.9)
	5	38/62 (61.3)	27/62 (43.5)
	6	12/33 (36.4)	21/33 (63.6)
	7	10/18 (55.6)	9/18 (50.0)
	8	16/29 (55.2)	11/29 (37.9)
	9	16/23 (69.6)	5/23 (21.7)
	10	19/32 (59.4)	14/32 (43.8)
	11	12/30 (40.0)	6/30 (20.0)
B	1	19/41 (46.3)	25/41 (61.0)
	2	20/47 (42.6)	29/47 (61.7)
	3	14/55 (25.5)	21/55 (38.2)
	4	14/30 (46.7)	13/30 (43.3)
	5	10/23 (43.5)	10/23 (43.5)
	6	21/35 (60.0)	14/35 (40.0)
	7	16/33 (48.5)	12/33 (36.4)
	8	19/35 (54.3)	17/35 (48.6)
	9	16/42 (38.1)	26/42 (61.9)
	10	20/25 (80.0)	14/25 (56.0)
	11	14/30 (46.7)	8/30 (26.7)
	12	9/37 (24.3)	9/37 (24.3)

### Statistical modelling

#### Infection with *Eimeria* spp. or *Campylobacter* spp.

An animal's age was a predictor of *Eimeria* infection, with infections being significantly more common in young animals (Table 5). There was a seasonal pattern in *Eimeria* infection rates, which were significantly higher in winter, spring and summer than in autumn. *Campylobacter* infection prevalence also varied significantly with season, being higher in winter and spring than in autumn.

**Table 5.** Parameter estimates and standard errors for GLM models

Model response	Final covariates	Estimates	*P*	Odds ratio
*Eimeria* infection	Age	−0.54 (0.23)	0.023	0.58
	Spring	0.60 (0.25)	0.019	1.81
	Summer	1.36 (0.24)	<0.001	3.88
	Winter	0.75 (0.26)	0.004	2.11
*Campylobacter* infection	Spring	0.66 (0.23)	0.004	1.93
	Winter	1.00 (0.22)	<0.001	2.71
*Eimeria* burden	*Campylobacter* infection	−0.37 (0.14)	0.013	
	Age	−1392 (0.13)	<0.001	
	Spring	0.94 (0.20)	<0.001	
	Summer	1.71 (0.18)	<0.001	
	Winter	0.08 (0.23)	0.728	
	Previous month's rainfall	0.01 (0.00)	<0.01	
*E. ovinoidalis* infection	*E. bakuensis* infection	0.7248 (0.15)	<0.001	2.06
	Farm A	0.4945 (0.16)	0.002	1.63
	*Campylobacter* infection	−0.3553 (0.16)	0.032	0.70
	Summer	0.7995 (0.18)	<0.001	2.22
	Winter	0.5966 (0.22)	0.008	1.81
*E. bakuensis* infection	*E. ovinoidalis* infection	0.7353 (0.14)	<0.001	2.08
	Farm A	−0.3251 (0.15)	0.031	0.72

Only statistically significant results are shown.

#### Infection with *E. ovinoidalis* or *E. bakuensis*

Coinfection appears to be an important predictor of an individual's probability of infection, with animals infected with *E. bakuensis* being significantly more likely to also be infected with *E. ovinoidalis*. However, infection with *Campylobacter* reduced the probability of an individual having *E. ovinoidalis* (Table 5). Season also affected infection probability for *E. ovinoidalis*, with increased infection in summer and winter compared to autumn. Interestingly, *E. ovinoidalis* infections were more likely at Farm A, whilst *E. bakuensis* infections were more likely at Farm B (Table 5).

#### *Eimeria* spp. burdens

We also observed seasonal variation in the intensity of *Eimeria* infections, with infections being significantly more intense in spring and summer than in autumn. *Eimeria* infection intensity also correlated with the amount of rainfall during the month prior to sampling. Interestingly, *Eimeria* infection intensity also correlated with *Campylobacter* infection; *Eimeria* was present at significantly lower intensities in co-infected animals than in those in which only *Eimeria* was detected (Table 5).

## Discussion

This study explored the epidemiology of *Eimeria* and *Campylobacter* infections in flocks of naturally infected sheep in Cumbria, UK, cataloguing their diversity and charting their temporal dynamics over a 14-month period. Overall, the epidemiology of *Eimeria* infections in our study population appeared to be ‘typical’ in that, in keeping with previous studies, we observed that (i) the majority of animals shed *Eimeria* oocysts throughout the duration of the survey, (ii) *E. ovinoidalis* was the most prevalent (of 10) ovine-associated *Eimeria* species encountered, (iii) infection rates and intensities were more common in younger animals and (iv) infection rates and intensities varied with season (e.g. Joyner *et al*., 1966; O'Callaghan *et al*., 1987; Dittmar *et al*., 2010; de Souza *et al*., 2015; Carrau *et al*., 2018). The relative high frequency with which we encountered *E. bakuensis* was noteworthy, although geographical variation in the relative abundance of ovine-associated *Eimeria* species is recognized while far from fully explored (de Souza *et al*., 2015; Carrau *et al*., 2018). The basis of such variation is unclear, although adaptation of different species to climatic conditions has been proposed (de Souza *et al*., 2015; Carrau *et al*., 2018). However, this proposal included the association of *E. ovinoidalis* to dry arid climates, which is at odds with our observation of this species at high prevalence in North West England, where precipitation levels are amongst the highest in Europe (https://www.eea.europa.eu/data-and-maps/figures/average-annual-precipitation). Other possibilities include variation in the rate and efficiency of sporulation, as has been described for some avian infecting eimerians (Norton and Chard, 1983), possibly biasing efficient replication by different *Eimeria* species under different environmental conditions.

Similarly, the epidemiology of *Campylobacter* infections was also akin to that described in previous surveys; our overall infection prevalence estimates of 32.6% on Farm A and 29.1% on Farm B are in close agreement with the findings of other surveys of sheep in the UK (Stanley and Jones, 2003; Grove-White *et al*., 2010; Sproston *et al*., 2011), as is our observation of a clear seasonal variation in infection prevalence. However, whereas others have reported higher infection prevalence in spring and summer (Stanley and Jones, 2003; Grove-White *et al*., 2010), in our survey, animals were more likely to be infected in winter and spring. This discrepancy may reflect differences in husbandry/farm management practices, which have been shown to influence *Campylobacter* shedding rates (Grove-White *et al*., 2010).

Interestingly, we observed that the intensity of *Eimeria* oocyst shedding (but not their prevalence) was significantly lower in animals co-infected with *Campylobacter* than in animals not co-infected with *Campylobacter*. This observation suggests that either the presence of *Campylobacter* leads to a reduction in *Eimeria* infection intensity or that higher *Eimeria* infection intensities impede *Campylobacter* colonization of the intestine. Previous work suggests both alternatives are feasible. Bacterial interference of *Eimeria* activity has been observed *in vitro*, with secretions from *Lactobacillus* species being shown to impede *Eimeria tenella* invasion of cultures of Madin-Darby bovine kidney cells (Tierney *et al*., 2004). Furthermore, the ability of bacteria to interfere with the development of apicomplexan oocyst development (*in vitro*) has been demonstrated for *Cryptosporidium* (Deng *et al*., 2001). The impact of parasite infections on the composition and activity of the microbiome is now well accepted based on studies of experimental (Hayes *et al*., 2010; Cantacessi *et al*., 2014) and naturally acquired (Kreisinger *et al*., 2015) infections. These studies include several that have explored the interaction between coccidian parasites and the microbiome; evidence of intestinal coccidial infection-induced perturbation of the microbiome has been reported in BALB/c mice (Huang *et al*., 2018*a*) and chickens (Macdonald *et al*., 2017; Huang *et al*., 2018*b*). Perhaps of most relevance to our study are observations drawn from experimental co-infection of poultry with *Eimeria* parasites and various enteric bacterial pathogens. The presence of *Eimeria* was found to enhance the growth of *Clostridium perfringens* and *Salmonella* Typhimurium in experimentally infected chickens (Arakawa *et al*., 1981; Collier *et al*., 2008) and, very recently, Macdonald *et al*. (2019) observed that increased fecal shedding of *C. jejuni* was associated with concomitant *E. tenella* infection in chickens. The results of one trial in this study indicated that fecal shedding rates of *E. tenella* oocysts were not affected by the presence of *C. jejuni*, whereas in another trial, *C. jejuni* infection intensities in the caeca and cloaca were significantly higher in birds co-infected with a higher dose of *E. tenella* than birds which received 10 times fewer parasites. The disparity between these observations and our results is noteworthy, since the association between *E. tenella* infection and increased *C. jejuni* load was detected during pathogenic parasite challenge that incurred notable haemorrhagic pathology. The increased mucus secretion and haemorrhage caused by concurrent pathogenic *E. tenella* challenge of chickens likely served to provide a source of nutrients and iron in support of *C. jejuni* proliferation (Palyada *et al*., 2004; Van Deun *et al*., 2008). The lower levels of *Eimeria* oocyst shedding described herein suggest a parasite–host system in enzootic stability, lacking such pathologies. Exploration of our findings in an aptly designed experimental model of sub-clinical and clinical disease would be worthwhile.

The analyses of *Eimeria* species level interactions provide another possible explanation for the observed lower intensity of *Eimeria* (genus) infections in animals co-infected with *Campylobacter*. These analyses indicated that infection with *Campylobacter* reduced the probability of an individual having *E. ovinoidalis* but did not significantly affect the probability of an individual having *E. bakuensis*. Thus, it may be that *Campylobacter* interacts with some but not all *Eimeria* species and that the observed loss of *Eimeria* infection intensity resulted from a reduction in the diversity of co-infecting *Eimeria* species.

Coinfection by different *Eimeria* species was also an important predictor of an individual's probability of infection. A sheep infected with *E. bakuensis* was significantly more likely to also be infected with *E. ovinoidalis* and *vice-versa*. Mixed infections with two or more *Eimeria* species are the norm for many reservoir hosts, including sheep, but as yet little work has been reported exploring competitive or facilitative interactions between species. Seville *et al*. (1996) surveyed infections by six *Eimeria* species in Wyoming ground squirrels (*Spermophilus elegans*) and reported several positive interactions between species but found no evidence for inter-species competition. Similarly, positive interaction was reported between co-infecting *Eimeria* species in red squirrels (*Sciurus vulgaris*) (Bertolino *et al*., 2003) and guanacos (*Lama guanicoe*) (Moreno *et al*., 2013). All authors proposed that these positive correlations reflected shared transmission pathways for different species, an explanation that may well also be valid for our observations. Conversely, other groups have reported apparent competition between co-infecting *Eimeria* species, based on surveys of co-infections in lizards (*Phelsuma ornate*) (Leinwand *et al*., 2005) and rabbits (*Sylvilagus floridanus*) (Bertolino *et al*., 2010).

In conclusion, the epidemiologies we quantified for *Eimeria* and *Campylobacter* infections in sheep align well with those described previously, and we confirm the influence of previously established epidemiological determinants. However, the concurrent survey of both taxa has revealed interactions between co-infectors that potentially affect the transmission dynamics of both. The importance of such interactions is well recognized among parasite ecologists (Telfer *et al*., 2010), but they are also clearly relevant to applied veterinary science given the strong correlation between intestinal microbiome structure, feed efficiency and thus livestock productivity (Stanley *et al*., 2012; Patil *et al*., 2018). Finally, our work underlines the value of livestock as models for studying the dynamics of natural parasite communities, particularly animals, such as the sheep we worked with, that graze all year round on rough pasture. Each sheep can be easily identified, and losses are low, so long-term repeated sampling of individuals is straight-forward. Furthermore, reagents for quantification of numerous biomarkers are commercially produced, indicators of fitness (productivity) are well established, and medicines of proven efficacy against specific parasites are available for intervention studies.
